# Symptoms and problems reported by patients with non-cancer diseases through open-ended questions in specialist palliative care: a national register-based study

**DOI:** 10.1007/s00520-024-08345-1

**Published:** 2024-02-02

**Authors:** Leslye Rojas-Concha, Maiken Bang Hansen, Mogens Groenvold

**Affiliations:** 1 Palliative Care Research Unit, Department of Geriatrics and Palliative Medicine GP, Bispebjerg and Frederiksberg Hospitals, University of Copenhagen, Bispebjerg Bakke 23, DK-2400 Copenhagen, NV Denmark; 2 Section for Health Services Research, Department of Public Health, University of Copenhagen, Copenhagen, Denmark

**Keywords:** Palliative care, Prevalence, Cardiovascular diseases, Neurological diseases, Lung diseases and kidney diseases

## Abstract

**Purpose:**

Since 2010, a comprehensive symptom/problem (S/P) assessment has been carried out in Danish specialist palliative care using the EORTC QLQ-C15-PAL questionnaire and the open-ended “Write In three Symptoms/Problems” (WISP) instrument. On WISP patients can report up to three S/Ps not included in the EORTC QLQ-C15-PAL. However, little is known about which S/Ps patients with non-cancer diseases report using WISP. Therefore, we investigated the prevalence and severity of S/Ps reported on WISP by non-cancer patients in specialist palliative care and compared these S/Ps with those previously reported by cancer patients.

**Methods:**

This register-based study collected data from the Danish Palliative Care Database. We included adult patients with non-cancer diseases answering the EORTC QLQ-C15-PAL at admittance to specialist palliative care between 2016 and 2021. WISP responses were qualitatively categorized, and their prevalence and severity calculated.

**Results:**

Of the 2323 patients with non-cancer diseases answering the EORTC QLQ-C15-PAL, 812 (34.9%) reported at least one S/P using WISP. A total of 1340 S/Ps were reported on WISP, of which 56.7% were not included in the EORTC QLQ-C15-PAL (i.e., were new). Edema, existential problems, dizziness, cough, and dysphagia were the most prevalent new S/Ps. Overall, 88.7% of the S/Ps were scored as moderate-severe. The prevalence of S/Ps reported on WISP did not significantly differ between cancer and non-cancer patients, except for existential problems, dysphagia, myoclonus, speaking problems, sweats, and vomiting.

**Conclusion:**

The similarities and differences in the prevalence of the most common S/Ps reported on WISP confirm that WISP improves symptom assessment regardless of patient diagnosis.

**Supplementary information:**

The online version contains supplementary material available at 10.1007/s00520-024-08345-1.

## Introduction

Palliative care aims to improve the quality of life of patients with life-threatening diseases through early assessment and control of their multiple symptoms and problems (S/Ps) [1]. Historically, palliative care has been developed primarily with a focus on cancer patients, but the growing aging population and changes in the prevalence of chronic diseases have increased the demand for palliative care in patients with diseases other than cancer [2]. Despite this high demand, several European countries have reported that a low proportion of patients with non-cancer diseases have access to specialist palliative care (less than 10%) and there is limited knowledge about their unmeet palliative care needs [3, 4].

Palliative care needs can be systematically assessed with patient-reported outcome (PRO) instruments, allowing patients to directly report their symptoms without interpretation from clinicians [5]. Among the most common PRO instruments used in palliative care are the European Organisation for Research and Treatment of Cancer Quality of Life Questionnaire Core 15 Palliative Care (EORTC QLQ-C15-PAL), the Edmonton Symptom Assessment Scale (ESAS), and the Memorial Symptom Assessment Scale (MSAS)[6].

However, standard PRO instruments may not measure all symptoms experienced by patients receiving palliative care. Therefore, a comprehensive symptom assessment has been carried out in specialist palliative care in Denmark by using the EORTC QLQ-C15-PAL (standard questionnaire) [7] in combination with the “Write In three Symptoms/Problems” (WISP) instrument. WISP is an open-ended question that permits patients to report up to three additional S/Ps not included in the EORTC QLQ-C15-PAL and to rate their severity [8]. These two instruments for symptom assessment of patients in specialized palliative care have been a national quality standard since 2010. Previous studies using WISP have shown that edema, dizziness, cough, sweats, and diarrhea are among the most prevalent additional S/Ps reported on WISP by cancer patients [8, 9], but little is known about which S/Ps patients with non-cancer diseases report using WISP.

This study aims to investigate the prevalence and severity of S/Ps reported on WISP by patients with non-cancer diseases, and to compare these S/Ps with those reported on WISP by cancer patients in Danish specialist palliative care. Data on cancer patients was previously published [8].

## Methods

### Patients and data

In this national register-based study, data was collected from the Danish Palliative Care Database, which contains clinical and demographic information on all patients referred to the 43 specialist palliative care services in Denmark since 2010. Clinicians in each service collect patient information on paper, which is then entered into a web-based system called the “Clinical Measurement System” and delivered to the Danish Palliative Care Database [10].

Inclusion criteria for patients in this study were (1) admission to palliative care between 1 January 2016 and 31 December 2021, (2) having a non-cancer disease, (3) being at least 18 years old, and (4) complete the EORTC QLQ-C15-PAL at the first day of contact with the palliative service or up to 3 days before.

Information on gender, age, having children, residence, cohabitation status, diagnosis, type of first contact, type of specialist palliative care service, and S/Ps reported on WISP were extracted from the Danish Palliative Care Database.

### Setting

In Denmark, specialist palliative care is provided in palliative teams in hospitals and hospices by physicians, nurses, and other professionals, e.g., psychologists [11]. By 2021, there were 43 specialist palliative care services, 24 palliative care teams in hospitals and 19 hospices. Of the 24 palliative care teams, 5 treated inpatients and outpatients and 19 treated only outpatients [12]. About 4% of patients admitted to specialist palliative in Denmark have diagnoses other than cancer [10, 12].

### Symptom assessment

The EORTC QLQ-C15-PAL questionnaire is an abbreviated version of the EORTC QLQ-C30 developed for patients in palliative care. It contains 15 items assessing the severity of 10 symptoms/functions: physical function, emotional function, pain, fatigue, nausea, dyspnea, sleeping difficulties, appetite loss, constipation, and overall quality of life. Symptoms/functions are rated as 1 (not at all), 2 (a little), 3 (quite a bit), or 4 (very much) and overall quality of life from 1 (very poor) to 7 (excellent) [7].

The WISP instrument supplements the EORTC QLQ-C15-PAL by allowing patients to report up to three additional S/Ps (through open-ended responses) after completing the EORTC QLQ-C15-PAL with the same 1-week timeframe. The additional symptoms severity are rated using the same response categories as on the EORTC QLQ-C15-PAL, i.e., from 1 (not at all) to 4 (very much) [8].

This study only reports S/Ps listed on WISP.

### Data analyses

Sociodemographic and clinical characteristics were summarized as proportions. Chi-square test was used to compare the characteristics of patients who did or did not report S/Ps using WISP (significance level 0.05).

WISP responses were analyzed qualitatively and quantitatively. We defined a patient as having a symptom/problem on WISP if it was rated at least “2 (a little).” All WISP responses rated 2, 3, or 4 were coded using a list of 61 symptom/problem categories developed for this instrument in a previous study [8], and new codes could be added if relevant.

The prevalence of each symptom/problem category was calculated as an “absolute proportion” in order to be comparable with the symptom/problem prevalences reported by cancer patients in a previously published study [8]. Thus, the prevalence was calculated for all patients answering the EORTC QLQ-C15-PAL overall and by diagnosis group (cardiovascular, neurological, lung, kidney, and other non-cancer disease). The severity was calculated as the proportion of S/Ps reported as “a little” (mild), “quite a bit” (moderate), and “very much” (severe).

We also calculated the mean and median number of S/Ps reported for two populations: all patients with non-cancer diseases answering the EORTC QLQ-C15-PAL and only those who reported S/Ps using WISP.

The prevalence of the 15 most frequent new S/Ps reported on WISP by cancer and non-cancer patients was compared using Fisher’s exact test. Data analyses were conducted using Statistical Package for the Social Sciences version 25.

## Results

### Study population

From the 2323 patients with non-cancer diseases completing the EORTC QLQ-C15-PAL at admittance to specialist palliative care between 1 January 2016 and 31 December 2021, 812 (34.9%) reported at least one symptom using the WISP instrument.

Table 1 compares the sociodemographic and clinical characteristics of patients with non-cancer diseases who reported or did not report a symptom using WISP. Comparisons between the two groups of patients showed that the distribution of patient characteristics was not significantly different, except for diagnosis and type of first contact and type of palliative care service. The largest proportion of patients reporting S/Ps on WISP had a lung disease, were outpatients, and received treatment from a palliative care team. More details can be seen in Table 1.

**Table 1:** Characteristics of patients answering the EORTC QLQ-C15-PAL questionnaire who reported or did not report symptoms and problems on the WISP instrument

Characteristics	Reported symptoms and problems on WISP				
	Yes (*n* = 812)		No (*n* = 1511)		
	*N*	%	*N*	%	*p* value
Gender					0.259
Female	388	47.8	685	45.3	
Male	424	52.2	826	54.7	
Age (years)					0.192
18–39	23	2.8	46	3.0	
40–49	26	3.2	61	4.0	
50–59	83	10.2	117	7.7	
60–69	185	22.8	312	20.6	
70–79	314	38.7	602	39.8	
80 +	181	22.3	373	24.7	
Having children					0.415
No children	120	14.8	191	12.6	
Children, at least one younger than 18 years	35	4.3	68	4.5	
Children, all at least 18 years old	624	76.8	1200	79.4	
Unknown	33	4.1	52	3.4	
Residence					0.770
Private (flat, house, etc.)	744	91.6	1372	90.8	
Nursing home/senior residence	58	7.1	117	7.7	
Other	3	0.4	10	0.7	
Unknown	7	0.9	12	0.8	
Cohabitation status					0.698
Living alone	293	36.1	591	39.1	
Living with spouse/partner	398	49.0	705	46.7	
Living with children	16	2.0	20	1.3	
Living with spouse/partner and children	20	2.5	33	2.2	
Living with parents	5	0.6	7	0.5	
Living with others	5	0.6	9	0.6	
Unknown	75	9.2	146	9.7	
Diagnosis					0.016
Cardiovascular disease	133	16.4	299	19.8	
Neurological disease (not stroke)	155	19.1	212	14.0	
Lung disease	337	41.5	643	42.6	
Kidney disease	70	8.6	130	8.6	
Other non-cancer disease	117	14.4	227	15.0	
Type of first contact					0.006
Outpatient	529	65.1	1068	70.7	
Inpatient	283	34.9	443	29.3	
Type of service					0.011
Palliative care team	503	61.9	1016	67.2	
Hospice	309	38.1	495	32.8	

### Symptom prevalence

**Fig. 1:**
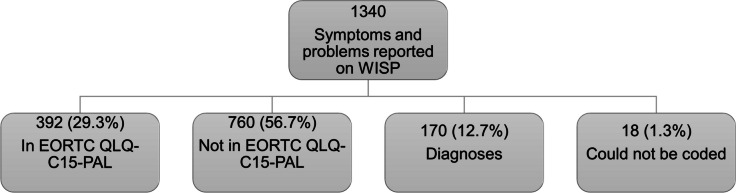
Classification of WISP responses reported by 812 patients with non-cancer diseases

In total, 1340 S/Ps were reported on WISP, of which 56.7% were not included in the EORTC QLQ-C15-PAL (i.e., were new), 29.3% were already included in the EORTC QLQ-C15-PAL, 12.7% were diagnoses, and 1.3% could not be coded due to misspelling or incomplete information (Fig. 1). WISP responses coded as “diagnoses” are described in the Supplementary Table 1, and among the most frequent diagnoses reported were mucus (22.9%), medication problems (9.4%), and heart problems (7.1%). Diagnoses and responses that could not be coded were excluded.

The 1152 S/Ps included or not in the EORTC QLQ-C15-PAL were classified into 57 symptom/problem categories (Table 2). The 2323 patients with non-cancer diseases answering the EORTC QLQ-C15-PAL reported on average 0.6 S/Ps on WISP with a median of 0 (range 0–3), whereas the 812 patients who reported S/Ps using WISP reported on average 1.7 S/Ps with a median of 1 (range 1–3). Overall, the most prevalent new S/Ps reported on WISP in decreasing order were edema, existential problems, dizziness, cough, and dysphagia. Edema (2.9–3.2%) and existential problems (3.0–3.8%) were the most prevalent S/Ps reported by patients with lung and other non-cancer diseases, respectively. Dizziness (3.2–4.0%) was more prevalent in patients with cardiovascular and kidney disease, whereas cough (2.7%) and dysphagia (5.2%) were more prevalent in patients with neurological diseases, as shown in Table 2.

**Table 2:** Prevalence of 57 symptoms and problems reported on the WISP instrument among 2323 patients with non-cancer diseases who answered the EORTC QLQ-C15-PAL. Symptoms and problems already included in the EORTC QLQ-C15-PAL are in italic

57 symptom/problem categories	All patients answering the EORTC QLQ-C15-PAL *N* = 2323		Patients divided by diagnosis group									
			Cardiovascular disease *N* = 432		Neurological disease (excluding stroke) *N* = 367		Lung disease *N* = 980		Kidney disease *N* = 200		Other non-cancer diseases *N* = 344	
	*N*	%	*N*	%	*N*	%	*N*	%	*N*	%	*N*	%
*Impaired emotional function*	132	5.7	20	4.6	16	4.4	71	7.2	7	3.5	18	5.2
*Impaired physical function*	83	3.6	9	2.1	30	8.2	25	2.6	7	3.5	12	3.5
*Pain*	76	3.3	10	2.3	11	3.0	30	3.1	9	4.5	16	4.7
*Dyspnea*	66	2.8	9	2.1	7	1.9	40	4.1	2	1.0	8	2.3
Edema	63	2.7	12	2.8	8	2.2	28	2.9	4	2.0	11	3.2
Existential problems	62	2.7	11	2.5	4	1.1	29	3.0	5	2.5	13	3.8
Dizziness	59	2.5	14	3.2	8	2.2	27	2.8	8	4.0	2	0.6
Cough	44	1.9	4	0.9	10	2.7	22	2.2	1	0.5	7	2.0
Dysphagia	44	1.9	12	2.8	19	5.2	11	1.1	0	0.0	2	0.6
Diarrhea	29	1.2	5	1.2	8	2.2	7	0.7	5	2.5	4	1.2
Vision problems	28	1.2	3	0.7	5	1.4	11	1.1	6	3.0	3	0.9
Myoclonus^a^	28	1.2	2	0.5	8	2.2	7	0.7	5	2.5	6	1.7
*Fatigue*	27	1.2	5	1.2	7	1.9	11	1.1	2	1.0	2	0.6
Dry mouth	27	1.2	5	1.2	4	1.1	14	1.4	3	1.5	1	0.3
Sore mouth	24	1.0	4	0.9	2	0.5	13	1.3	0	0.0	5	1.5
Speaking problems	24	1.0	3	0.7	14	3.8	6	0.6	0	0.0	1	0.3
Itching	23	1.0	4	0.9	3	0.8	4	0.4	6	3.0	6	1.7
Incontinence^b^	21	0.9	4	0.9	5	1.4	8	0.8	2	1.0	2	0.6
Shakiness	19	0.8	0	0.0	3	0.0	8	0.8	2	1.0	6	1.7
Numbness/tingling	19	0.8	4	0.9	4	1.1	9	0.9	2	1.0	0	0.0
Indigestion	17	0.7	2	0.5	2	0.5	8	0.8	1	0.5	4	1.2
Confusion	15	0.6	4	0.9	3	0.8	7	0.7	0	0.0	1	0.3
Urinary problems	14	0.6	4	0.9	1	0.3	6	0.6	3	1.5	0	0.0
Reduced memory	14	0.6	3	0.7	1	0.3	6	0.6	1	0.5	3	0.9
Headache	13	0.6	2	0.5	3	0.8	5	0.5	1	0.5	2	0.6
Social problems	13	0.6	1	0.2	4	1.1	2	0.2	3	1.5	3	0.9
Hypersalivation	12	0.5	1	0.2	7	1.9	3	0.3	0	0.0	1	0.3
Hearing problems	12	0.5	3	0.7	3	0.8	4	0.4	2	1.0	0	0.0
Skin problems	12	0.5	1	0.2	4	1.1	4	0.4	0	0.0	3	0.9
*Sleeping difficulties*	11	0.5	3	0.7	1	0.3	5	0.5	0	0.0	2	0.6
Distress in the body	10	0.4	4	0.9	2	0.5	2	0.2	0	0.0	2	0.6
Vomiting	9	0.4	3	0.7	1	0.3	2	0.2	1	0.5	2	0.6
Bleeding	9	0.4	2	0.5	1	0.3	4	0.4	0	0.0	2	0.6
Sweats	9	0.4	1	0.2	4	1.1	2	0.2	0	0.0	2	0.6
Hallucinations^c^	8	0.3	1	0.2	0	0.0	5	0.5	0	0.0	2	0.6
*Lack of appetite*	7	0.3	2	0.5	2	0.5	2	0.2	1	0.5	0	0.0
Bloating	6	0.3	1	0.2	2	0.5	1	0.1	1	0.5	1	0.3
*Nausea*	5	0.2	1	0.2	0	0.0	0	0.0	0	0.0	4	1.2
Taste change	5	0.2	2	0.5	2	0.5	1	0.1	0	0.0	0	0.0
Heaviness	5	0.2	1	0.2	0	0.0	1	0.1	2	1.0	1	0.3
Other eye symptoms	4	0.2	1	0.2	0	0.0	1	0.1	0	0.0	2	0.6
Other ear problems	4	0.2	0	0.0	0	0.0	2	0.2	1	0.5	1	0.3
Palpitations	4	0.2	0	0.0	1	0.3	3	0.3	0	0.0	0	0.0
Concentration problems	4	0.2	0	0.0	1	0.3	2	0.2	0	0.0	1	0.3
Cognitive dysfunction	3	0.1	0	0.0	0	0.0	1	0.1	2	1.0	0	0.0
Bad dreams	3	0.1	0	0.0	0	0.0	1	0.1	1	0.5	1	0.3
*Constipation*	3	0.1	1	0.2	0	0.0	0	0.0	0	0.0	2	0.6
Hoarseness	3	0.1	2	0.5	1	0.3	0	0.0	0	0.0	0	0.0
Weight loss	3	0.1	1	0.2	1	0.3	0	0.0	0	0.0	1	0.3
Housing problems	3	0.1	0	0.0	0	0.0	2	0.2	0	0.0	1	0.3
Burning sensation	3	0.1	0	0.0	2	0.5	1	0.1	0	0.0	0	0.0
Easy to tears	2	0.1	0	0.0	2	0.5	0	0.0	0	0.0	0	0.0
Heartburn	2	0.1	0	0.0	0	0.0	2	0.2	0	0.0	0	0.0
Hiccup	2	0.1	0	0.0	0	0.0	1	0.1	1	0.5	0	0.0
Fever	2	0.1	1	0.2	0	0.0	0	0.0	0	0.0	1	0.3
Economic problems	2	0.1	0	0.0	0	0.0	0	0.0	0	0.0	2	0.6
Thirst	1	0.0	1	0.2	0	0.0	0	0.0	0	0.0	0	0.0
Total	1152	100	189	16.4	227	19.7	467	40.5	97	8.4	172	1.9

Prevalences were calculated as absolute proportions to be comparable with prevalences reported by cancer patients in a previously published study

^a^Including muscle cramps and spasms

^b^Including urinary, stool, and unspecified incontinence

^c^Including visual, auditory and unspecified hallucinations

The most prevalent S/Ps already included in the EORTC QLQ-C15-PAL reported by all patients with non-cancer diseases were impaired emotional function, impaired physical function, pain, and dyspnea. The 132 problems coded as impaired emotional function included feeling anxious (62.9%), irritable (13.6%), concerned (9.1%), stressed (8.3%), and sad (6.1%). Problems coded as impaired physical function (*n* = 83) included reduced mobility (43.3%), balance/coordination problems (19.3%), difficulties to change positions (14.5%), walking problems (13.3%), and muscular weakness (9.6%).

### Symptom severity

Supplementary Table 2 shows the frequency and severity of the 57 S/Ps reported on WISP. Overall, most of the S/Ps were rated as severe (54.1%) or moderate (34.6%), and very few S/Ps were rated as mild (11.3%). The most frequent S/Ps reported as severe were economic problems (100%), housing problems (100%) and hallucinations (87.5%), whereas the most frequent S/Ps reported as mild were heartburn (100%), constipation (66.7%), and hiccup (50.0%).

### Comparison of symptom prevalence

**Fig. 2:**
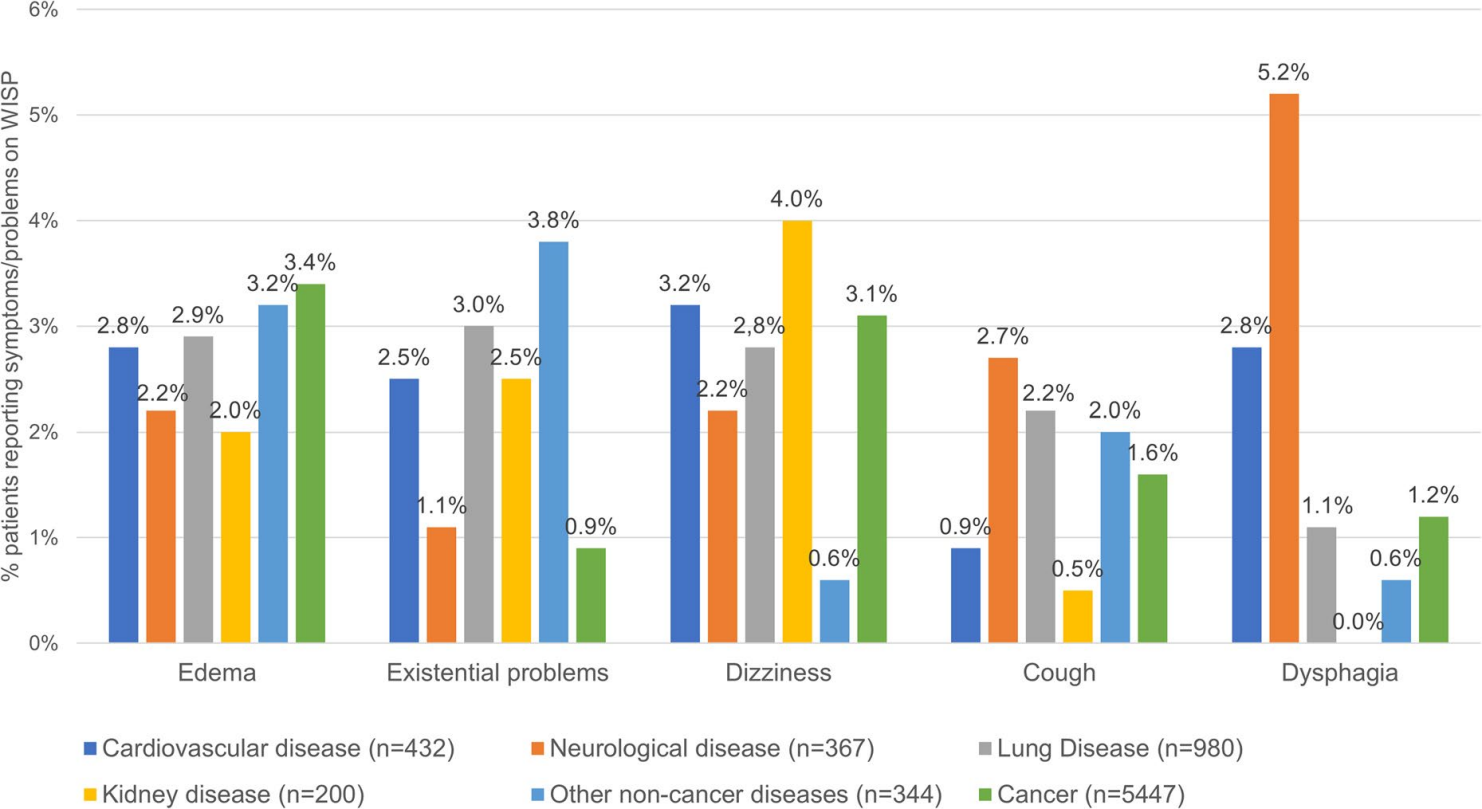
Prevalence of the 5 most frequent symptoms and problems reported on WISP according to diagnosis group, among 2323 non-cancer patients and 5447 cancer patients

The prevalence of the most common new S/Ps reported on WISP was similar among patients with non-cancer diagnoses and those with cancer completing the EORTC QLQ-C15-PAL at admittance to specialist palliative care (Fig. 2). Edema was the most prevalent new S/P reported in both populations (≥ 3%), while thirst was among the least prevalent (≤ 0.2%) (Table 3).

**Table 3:** Prevalence of the 15 most frequent new symptoms and problems reported on WISP by non-cancer and cancer patients

Symptom/problem categories	Prevalence in 2323 **non-cancer patients** answering the EORTC QLQ-C15-PAL		Prevalence in 5447 **cancer patients** answering the EORTC QLQ-C15-PAL		
	*N*	%	*N*	*%*	*p* value
Edema	63	2.7	183	3.4	0.203
Dizziness	59	2.5	169	3.1	0.187
Existential problems	62	2.7	50	0.9	< 0.001
Cough	44	1.9	85	1.6	0.288
Sweats	9	0.4	80	1.5	< 0.001
Diarrhea	29	1.2	74	1.4	0.746
Dysphagia	44	1.9	65	1.2	0.020
Dry mouth	27	1.2	73	1.3	0.583
Incontinence	21	0.9	73	1.3	0.113
Vision problems	28	1.2	63	1.2	0.908
Sore mouth	24	1.0	69	1.3	0.426
Myoclonus	28	1.2	32	0.6	0.007
Vomiting	9	0.4	66	1.2	< 0.001
Speaking problems	24	1.0	30	0.6	0.025
Numbness/tingling	19	0.8	56	1.0	0.448

The prevalence of symptoms and problems reported by cancer patients are from “Which symptoms and problems do advanced cancer patients admitted to specialized palliative care report in addition to those included in the EORTC QLQ-C15-PAL? A register-based national study” by Rojas-Concha et al. Support Care Cancer 2020. https://doi.org/10.1007/s00520-019-04976-x. Copyright © 2019, Springer-Verlag GmbH Germany, part of Springer Nature

Patients with non-cancer diseases had significantly higher prevalences of existential problems (2.7% vs. 0.9%), dysphagia (1.9% vs. 1.2%), myoclonus (1.2% vs. 0.6%), and speaking problems (1.0% vs. 0.6%). In contrast, cancer patients had significantly higher prevalences of sweats (1.5% vs. 0.4%) and vomiting (1.2% vs. 0.4%) (Table 3).

## Discussion

### Main findings

Roughly a third (34.9%) of the 2323 patients with non-cancer diseases answering the EORTC QLQ-C15-PAL between 2016 and 2021 reported at least one S/P on WISP. Edema, existential problems, dizziness, cough, and dysphagia were the most prevalent new S/Ps reported on WISP among patients with cardiovascular, neurological, lung, kidney, and other non-cancer diseases. Overall, 88.7% of the S/Ps reported on WISP were scored as moderate to severe and can therefore be considered bothersome. Patients with non-cancer diseases reported similar prevalence of S/Ps on WISP to that previously reported by cancer patients, except for higher prevalences of existential problems, dysphagia, myoclonus and speaking problems, and lower prevalences of sweats and vomiting.

### Comparison with previous literature

The WISP instrument identified several S/Ps experienced by patients with non-cancer diseases at admittance to specialist palliative care that were not covered by the EORTC QLQ-C15-PAL. Among the most prevalent new S/Ps listed on WISP, edema, dizziness, cough, and dysphagia have also previously been reported as frequent symptoms experienced by patients with non-cancer diseases receiving palliative care [13–15].

In this study, edema was the most prevalent new S/P listed on WISP, especially by patients with lung (2.9%) and other non-cancer diseases (3.2%), which may be explained by different causes of edema (or lymphoedema) such as venous hypertension and hypoalbuminemia frequently observed in patients with advanced liver, heart, and lung conditions [13]. Existential problems (particularly having thoughts about death, feeling lonely, hopeless, and/or powerless) were also prevalent in patients with lung (3.0%) and other non-cancer diseases (3.8%). This is in agreement with an earlier study comparing existential distress in four non-cancer populations receiving palliative care, where the proportion reporting general dissatisfaction and hopelessness was higher among patients with obstructive pulmonary disease (COPD) (> 5%) than among patients with neurological and kidney diseases [16]. On the other hand, dizziness was most prevalent in patients with cardiovascular (3.2%) and kidney diseases (4.0%), which is in line with a systematic review showing that dizziness is a prevalent symptom reported by patients with chronic heart and renal failure at the end of life (> 21%) [14]. Cough and dysphagia were prevalent symptoms listed by patients with neurological diseases (2.7–5.2%). This is consistent with previous studies showing that difficulties swallowing, persistent coughing or aspiration are commonly caused by underlying neurological disorders such as Parkinson’s disease, multiple sclerosis, or dementia, where particularly dysphagia becomes more severe in the palliative phase near death [15, 17].

In line with previous studies collecting symptoms through open-ended questions in palliative care patients, 88.7% of S/Ps reported via WISP by patients with non-cancer diseases were rated as moderate to severe (score 3–4), highlighting that patients voluntarily report symptoms when they perceive them to be distressing [8, 18, 19].

Several studies have compared the symptomatology of cancer and non-cancer patients in palliative care using PRO instruments or chart reviews [20–25], but information of which S/Ps non-cancer patients report using open-ended questions or how these S/Ps differ from those reported by cancer patients is limited. A recent study including 102 patients with advanced chronic heart failure examined the symptoms reported via open-ended questions followed by closed questions using the Palliative care Outcome Scale (POS/IPOS) and found that poor mobility (24%), shortness of breath (16%), fatigue (14%), and pain (10%) were among the most frequent symptoms reported using open-ended questions [26].

In our study, we found that the prevalence of the most frequently S/Ps reported on WISP did not significantly differ between cancer and non-cancer patients, except for existential problems, dysphagia, myoclonus, speaking problems, sweats, and vomiting. In both studies, the same methodology was used to calculate the prevalences as “absolute proportions,” i.e., S/Ps reported on WISP among all patients who completed the EORTC QLQ-C15-PAL. Furthermore, non-cancer patients did not report new S/P categories on WISP (i.e., all the reported S/Ps fitted into one of the 61 S/P categories already developed for cancer patients) [8]. Although the EORTC QLQ-C15-PAL questionnaire was developed to access the most frequent S/Ps among cancer patients in palliative care [7], it also appears to include the most common S/Ps among non-cancer patients, since the frequency of extra S/Ps reported on WISP was similar for cancer and non-cancer patients.

Patients with non-cancer diseases completing the EORTC QLQ-C15-PAL on average reported 0.6 S/Ps on WISP, comparable to the average of 0.5 S/Ps previously reported by cancer patients [8], but slightly lower than the average of 1.3 S/Ps reported via open-ended question by 62 patients with chronic non-cancer pain [27]. In both studies, edema was the most prevalent new S/Ps listed on WISP and 49% of patients described it as severe. This is consistent with the literature showing that edema is one of the most common symptoms experienced by palliative care patients (5–10%) with increasing prevalence towards the end of life, especially in non-cancer patients [13, 23].

Both groups of patients (i.e., cancer and non-cancer patients) most frequently reported pain, impaired emotional function, and impaired physical function on WISP, even though they were already covered by EORTC QLQ-C15-PAL. One explanation for this is that patients needed to add more information about a S/P, for example, patients who reported pain always used WISP to describe its location or frequency they experienced pain. Similarly, many cancer and non-cancer patients listed anxiety (55–63%) as a frequent emotional impairment although included in the EORTC QLQ-C15-PAL. Balance/coordination problem was also frequently reported on WISP (19–26%) and these physical functioning problems are not asked directly in the standard questionnaire although it measures physical functioning.

There were some differences in the reporting of common S/Ps on WISP between cancer and non-cancer patients. Patients with non-cancer diseases reported a significantly higher prevalence of existential problems, dysphagia, myoclonus, and speaking problems than cancer patients. This high prevalence of existential problems is surprising since the literature has shown that cancer patients report high levels of existential distress, leading to poor quality of life and increased risk of suicide [28–30]. These findings may be explained by 42% of patients answering WISP in our study had a lung disease (mainly COPD) and a recent systematic review based on 35 selected articles concluded that existential distress significantly impact patients with COPD receiving palliative care [31]. The high prevalence of dysphagia and speaking problems seen in non-cancer patients with neurological diseases can be explained by the high frequency of swallowing disorders and speech motor disorders (i.e., dysarthria and apraxia) present in several neurological conditions such as Parkinson’s disease, cerebellar disease and multiple sclerosis [17, 32]. In addition, dysphagia is a frequently observed problem in patients with head, neck, and esophageal cancer due to the location of their primary tumor or after radiotherapy [33]; however, these patients only represented about 6% of the 1788 cancer patients reporting symptoms on WISP in the previous study [8], and thus had little impact on the prevalence of dysphagia reported on WISP by cancer patients overall. Finally, the high prevalence of myoclonus observed in patients with neurological and kidney diseases may be associated with renal failure and various neurodegenerative disorders like epilepsy and Parkinson’s disease causing this symptomatic and involuntary muscle activity [34, 35].

In comparison to patients with non-cancer diagnoses, those with cancer reported a significantly higher prevalence of sweats and vomiting. Night sweats and hot flashes have been frequently associated with endocrine therapy received by patients with prostate and breast cancer [36, 37], whereas vomiting is highly associated with cancer treatment and in with diagnoses such as gynecological and gastric cancer due to treatment and/or tumor progression [38, 39].

### Strengths and limitations

A strength in this study is that we analyzed a large national data set including 2323 patients with non-cancer diseases admitted to all specialist palliative care services in Denmark during 2016–2021 (of whom 812 reported S/Ps using WISP). Also, to our knowledge, this is the first large study investigating S/Ps reported via open-ended question by all patients with non-cancer diseases admitted to specialist palliative care and by diagnosis-related group (i.e., patients with cardiovascular, neurological, lung, kidney, and other non-cancer diseases), since most of the available studies in which patients in palliative care voluntarily report their symptoms through open-ended questions only include cancer patients [18, 19, 40]. However, although our study confirms that the use of WISP increases symptom recognition in specialized palliative care, the prevalence of S/Ps reported on WISP tends to be low as patients report fewer symptoms via open-ended questions than via systematic assessment, e.g., using PRO instruments. Furthermore, patients with non-cancer diseases have limited access to Danish specialist palliative care as in several other European countries [3, 4].

## Conclusions

This study confirmed the utility of the open-ended WISP question among patients with non-cancer diseases admitted to specialist palliative care in Denmark. The WISP which identified several S/Ps not measured by the standard EORTC questionnaire. Edema, existential problems, dizziness, cough, and dysphagia were among the most prevalent S/Ps reported on the open-ended WISP question.

There were similarities in the prevalence of the most common S/Ps reported on WISP across cancer and non-cancer patients but also a few distinct differences. This confirms that combining systematic symptom assessment and open-ended symptom reporting via WISP increases identification of palliative care needs, regardless patient diagnosis. Thus, we recommend the use of WISP in combination with the standard questionnaire in routine palliative care for a more comprehensive symptom assessment of patients.

### Supplementary information

Below is the link to the electronic supplementary material.

Supplementary file1 (DOCX 33 KB)

## Data Availability

The data utilized in this study are available through the Danish Palliative Care Database. Restrictions apply to the availability of these data.
