# Supplementary material for: Symptoms and problems reported by patients with non-cancer diseases through open-ended questions in specialist palliative care: a national register-based study
Source: Support Care Cancer. 2024 Feb 2;32(2):141. doi: 10.1007/s00520-024-08345-1 (PMC10837258; doi:10.1007/s00520-024-08345-1)
Supplement: Supplementary file 1 — Supplementary file1 (DOCX 33 KB) [file 520_2024_8345_MOESM1_ESM.docx]

Supplementary Table 1. Frequency of 170 responses listed on the WISP instrument and coded as diagnoses

| Diagnoses | N | % |
| --- | --- | --- |
| Mucus | 39 | 22.9 |
| Medication problems | 16 | 9.4 |
| Heart problems (not specified) | 12 | 7.1 |
| Pressure ulcer | 11 | 6.5 |
| Infection | 9 | 5.3 |
| Catheter problems | 8 | 4.7 |
| Respiratory diseases^a^ | 8 | 4.7 |
| Mental disorders^b^ | 6 | 3.5 |
| Ascites | 5 | 2.9 |
| Arthritis | 5 | 2.9 |
| Diabetes | 4 | 2.4 |
| Stiffness | 4 | 2.4 |
| Hernia | 4 | 2.4 |
| Neuritis | 4 | 2.4 |
| Renal problems | 3 | 1.8 |
| Hypotension | 3 | 1.8 |
| Intestinal obstruction | 3 | 1.8 |
| Fracture | 3 | 1.8 |
| Eczema or psoriasis | 2 | 1.2 |
| Restless legs disorder | 2 | 1.2 |
| Amyotrophic Lateral Sclerosis | 2 | 1.2 |
| Wound | 2 | 1.2 |
| Allergy | 2 | 1.2 |
| Fungus | 2 | 1.2 |
| Pulmonary edema | 2 | 1.2 |
| Hemorrhoid | 1 | 0.6 |
| Amputation | 1 | 0.6 |
| Parkinson | 1 | 0.6 |
| Spinal stenosis | 1 | 0.6 |
| Gout | 1 | 0.6 |
| Bad blood circulation | 1 | 0.6 |
| Cachexia | 1 | 0.6 |
| Apnea | 1 | 0.6 |
| Dementia | 1 | 0.6 |

^a^ Including: asthma, cold, pneumonia, and chronic obstructive pulmonary disease

^b^ Including: claustrophobia, panic, addiction problems and post-traumatic stress disorder

Supplementary Table 2. Frequency and severity of 57 symptoms and problems reported on the WISP instrument among 2,323 patients with non-cancer diseases who answered the EORTC QLQ-C15-PAL. Symptoms and problems already included in the EORTC QLQ-C15-PAL are in italic.

| 57 symptom/problem categories | Symptoms/problems reported on WISP N=1,152 | | | | | | | | | |
| --- | --- | --- | --- | --- | --- | --- | --- | --- | --- | --- |
|  | Frequency | | | Severity | | | | | | |
|  |  |  | Mild | | | Moderate | | Severe | |  |
|  | N | % | N | | % | N | % | N | % | |
| *Impaired emotional function* | 132 | 11.5 | 13 | | 9.8 | 46 | 34.8 | 73 | 55.3 | |
| *Impaired physical function* | 83 | 7.2 | 6 | | 7.2 | 14 | 16.9 | 63 | 75.9 | |
| *Pain* | 76 | 6.6 | 5 | | 6.6 | 35 | 46.1 | 36 | 47.4 | |
| *Dyspnea* | 66 | 5.7 | 1 | | 1.5 | 15 | 22.7 | 50 | 75.8 | |
| Edema | 63 | 5.5 | 13 | | 20.6 | 19 | 30.2 | 31 | 49.2 | |
| Existential problems | 62 | 5.4 | 7 | | 11.3 | 10 | 16.1 | 45 | 7.6 | |
| Dizziness | 59 | 5.1 | 10 | | 16.9 | 33 | 55.9 | 16 | 27.1 | |
| Cough | 44 | 3.8 | 6 | | 13.6 | 17 | 38.6 | 21 | 47.7 | |
| Dysphagia | 44 | 3.8 | 10 | | 22.7 | 18 | 40.9 | 16 | 36.4 | |
| Diarrhea | 29 | 2.5 | 4 | | 13.8 | 7 | 24.1 | 18 | 62.1 | |
| Vision problems | 28 | 2.4 | 2 | | 7.1 | 11 | 39.3 | 15 | 53.6 | |
| Myoclonus^a^ | 28 | 2.4 | 4 | | 14.3 | 12 | 42.9 | 12 | 42.9 | |
| *Fatigue* | 27 | 2.3 | 1 | | 3.7 | 8 | 29.6 | 18 | 66.7 | |
| Dry mouth | 27 | 2.3 | 2 | | 7.4 | 11 | 40.7 | 14 | 51.9 | |
| Sore mouth | 24 | 2.1 | 4 | | 16.7 | 10 | 41.7 | 10 | 41.7 | |
| Speaking problems | 24 | 2.1 | 1 | | 4.2 | 8 | 33.3 | 15 | 62.5 | |
| Itching | 23 | 2.0 | 4 | | 17.4 | 10 | 43.5 | 9 | 39.1 | |
| Incontinence^b^ | 21 | 1.8 | 0 | | 0.0 | 8 | 38.1 | 13 | 61.9 | |
| Shakiness | 19 | 1.6 | 2 | | 10.5 | 5 | 26.3 | 12 | 63.2 | |
| Numbness/tingling | 19 | 1.6 | 1 | | 5.3 | 10 | 52.6 | 8 | 42.1 | |
| Indigestion | 17 | 1.5 | 2 | | 11.8 | 8 | 47.1 | 7 | 41.2 | |
| Confusion | 15 | 1.3 | 3 | | 20.0 | 5 | 33.3 | 7 | 46.7 | |
| Urinary problems | 14 | 1.2 | 2 | | 14.3 | 7 | 50.0 | 5 | 35.7 | |
| Reduced memory | 14 | 1.2 | 2 | | 14.3 | 4 | 28.6 | 8 | 57.1 | |
| Headache | 13 | 1.1 | 2 | | 15.4 | 8 | 61.5 | 3 | 23.1 | |
| Social problems | 13 | 1.1 | 1 | | 7.7 | 4 | 30.8 | 8 | 61.5 | |
| Hypersalivation | 12 | 1.0 | 2 | | 16.7 | 3 | 25.0 | 7 | 58.3 | |
| Hearing problems | 12 | 1.0 | 0 | | 0.0 | 2 | 16.7 | 10 | 83.3 | |
| Skin problems | 12 | 1.0 | 3 | | 25.0 | 2 | 16.7 | 7 | 58.3 | |
| *Sleeping difficulties* | 11 | 1.0 | 1 | | 9.1 | 3 | 27.3 | 7 | 63.6 | |
| Distress in the body | 10 | 0.9 | 0 | | 0.0 | 5 | 50.0 | 5 | 50.0 | |
| Vomiting | 9 | 0.8 | 2 | | 22.2 | 3 | 33.3 | 4 | 44.4 | |
| Bleeding | 9 | 0.8 | 1 | | 11.1 | 4 | 44.4 | 4 | 44.4 | |
| Sweats | 9 | 0.8 | 4 | | 44.4 | 1 | 11.1 | 4 | 44.4 | |
| Hallucinations^b^ | 8 | 0.7 | 0 | | 0.0 | 1 | 12.5 | 7 | 87.5 | |
| *Lack of appetite* | 7 | 0.6 | 0 | | 0.0 | 1 | 14.3 | 6 | 85.7 | |
| Bloating | 6 | 0.5 | 0 | | 0.0 | 3 | 50.0 | 3 | 50.0 | |
| *Nausea* | 5 | 0.4 | 0 | | 0.0 | 2 | 40.0 | 3 | 60.0 | |
| Taste change | 5 | 0.4 | 1 | | 20.0 | 2 | 40.0 | 2 | 40.0 | |
| Heaviness | 5 | 0.4 | 0 | | 0.0 | 4 | 80.0 | 1 | 20.0 | |
| Other eye symptoms | 4 | 0.3 | 1 | | 25.0 | 1 | 25.0 | 2 | 50.0 | |
| Other ear problems | 4 | 0.3 | 0 | | 0.0 | 1 | 25.0 | 3 | 75.0 | |
| Palpitations | 4 | 0.3 | 0 | | 0.0 | 4 | 100 | 0 | 0.0 | |
| Concentration problems | 4 | 0.3 | 0 | | 0.0 | 3 | 75.0 | 1 | 25.0 | |
| Cognitive dysfunction | 3 | 0.3 | 0 | | 0.0 | 1 | 33.3 | 2 | 66.7 | |
| Bad dreams | 3 | 0.3 | 0 | | 0.0 | 2 | 66.7 | 1 | 33.3 | |
| *Constipation* | 3 | 0.3 | 2 | | 66.7 | 1 | 33.3 | 0 | 0.0 | |
| Hoarseness | 3 | 0.3 | 1 | | 33.3 | 1 | 33.3 | 1 | 33.3 | |
| Weight loss | 3 | 0.3 | 1 | | 33.3 | 1 | 33.3 | 1 | 33.3 | |
| Housing problems | 3 | 0.3 | 0 | | 0.0 | 0 | 0.0 | 3 | 100 | |
| Burning sensation | 3 | 0.3 | 0 | | 0.0 | 2 | 66.7 | 1 | 33.3 | |
| Easy to tears | 2 | 0.2 | 0 | | 0.0 | 1 | 50.0 | 1 | 50.0 | |
| Heartburn | 2 | 0.2 | 2 | | 100 | 0 | 0.0 | 0 | 0.0 | |
| Hiccup | 2 | 0.2 | 1 | | 50.0 | 0 | 0.0 | 1 | 50.0 | |
| Fever | 2 | 0.2 | 0 | | 0.0 | 1 | 50.0 | 1 | 50.0 | |
| Economic problems | 2 | 0.2 | 0 | | 0.0 | 0 | 0.0 | 2 | 100 | |
| Thirst | 1 | 0.1 | 0 | | 0.0 | 1 | 100 | 0 | 0.0 | |
| Total | 1,152 | 100 | 130 | | 11.3 | 399 | 34.6 | 623 | 54.1 | |

^a^ Including muscle cramps and spasms

^b^ Including urinary, stool, and unspecified incontinence

^c^ Including visual, auditory and unspecified hallucinations
